# Abdominal examination during pregnancy may enhance relationships between midwife, mother and child: a qualitative study of pregnant women’s experiences

**DOI:** 10.1186/s12884-023-05392-0

**Published:** 2023-01-31

**Authors:** Katrine Bruun Bonnén, Sara Marie Hebsgaard Offersen, Lea Høj Høstrup, Rikke Damkjær Maimburg

**Affiliations:** 1 Department of Clinical Medicine, Aarhus University, Palle Juul-Jensens Boulevard 82, 8200 Aarhus N, Denmark; 2 Steno Diabetes Center Aarhus, Aarhus University Hospital, Palle Juul-Jensens Boulevard 11, 8200 Aarhus N, Denmark; 3 User Perspectives and Community-based Interventions, Department of Public Health, University of Southern Denmark, J. B. Winsløws Vej 9, 5000 Odense C, Denmark; 4 Department of Midwifery, University College of Northern Denmark, Selma Lagerløfs Vej 2, 9220 Aalborg Ø, Denmark; 5 Department of Occupational Medicine, Danish Ramazzini Centre, Aarhus University Hospital, Palle Juul-Jensens Boulevard 99, 8200 Aarhus N, Denmark; 6 School of Nursing and Midwifery, Western Sydney University, Locked Bag 1797, Penrith, NSW 2751 Australia

**Keywords:** Abdominal examination, Midwifery, Maternity care, Attachment, Mind–body relations, Client–staff relations

## Abstract

**Background:**

Abdominal examination is a routine procedure performed by midwives several times during pregnancy to monitor the growth and well-being of the baby. Literature and instructions regarding abdominal examination focus on the technical performance, with limited attention paid to the women’s experience of the examination or the bonding-related aspects between the mother and baby. The aim of the study was to explore how pregnant women experience the abdominal examination and how the examination affects maternal–fetal attachment.

**Methods:**

Participant observation and semi-structured interviews with 10 pregnant women. We used thematic analysis to identify themes across the empirical material.

**Results:**

We identified the following four central themes: *an essential examination*, *the baby becomes real*, *the importance of being involved* and *different senses provide different experiences*. These themes describe how the women regarded the abdominal examination as an essential part of the midwifery consultation and considered it the occasion when the baby became real and tangible. Being prepared and involved before and during the examination were pivotal for how the examination was experienced by the women. The abdominal examination was crucial to the pregnant women because it provided them with important sensory aspects that were not obtained from ultrasound examination.

**Conclusion:**

The abdominal examination is regarded as essential in midwifery consultations and has the potential for supporting a woman’s bodily sensation of her baby, which is reinforced by the midwife’s manual palpation. Touch can be a way for a pregnant woman to become acquainted with her unborn child, which provides midwives a profound potential to facilitate the process of maternal–fetal attachment.

**Supplementary Information:**

The online version contains supplementary material available at 10.1186/s12884-023-05392-0.

## Background

Pregnant women undergo abdominal examination, which is a routine procedure, multiple times during pregnancy and birth. Midwives perform this examination through manual palpation, including auscultation of the fetal heartbeat. The abdominal examination is used to confirm and monitor the growth, well-being and positioning of the fetus. This is reflected in written notes of coded inscriptions representing the midwives’ physical assessment [1]. Medical instructions for abdominal examination focus on the technical measurements and performance of the midwife, paying limited or no attention to the experiential- or bonding-related aspects [2, 3].

In addition to revealing physical characteristics of the fetus, manual palpation may have the potential to improve prenatal attachment between the mother and her unborn child, which is also described as maternal–fetal attachment [4, 5]. Studies investigating maternal–fetal attachment deal with the extent of a mother’s feelings and behaviour regarding engagement with and affection towards her unborn child [6, 7]. A mother’s emotional bonding with her child is initiated before birth and is widely recognised as being of critical importance for the health and well-being of the child [6, 8, 9]. Furthermore, a high level of maternal–fetal attachment may support early childhood development in communication, motor and social domains [10]. Facilitating maternal–fetal attachment in the antepartum period is recommended to improve postnatal bonding [11] as well as maternal sensitivity, which is a mother’s ability to accurately perceive, interpret and respond appropriately to her infant’s signals and communications [12]. Moreover, a lack of maternal–fetal attachment is associated with maternal mental health issues, such as stress, anxiety and depression [11, 13], thus making maternal–fetal attachment especially important with regard to psychologically vulnerable mothers.

A positive bodily experience of being pregnant is also an important factor in terms of being attached to the unborn baby [14] and women may cope positively with body changes during pregnancy owing to events unique to pregnancy, such as increased perceived body functionality, positive social commentary and perceptual experiences such as feeling the baby kick [15]. However, there is lack of evidence on how the abdominal examination is experienced by the pregnant women and how the manual palpation and auscultation by the midwives affect the mother’s experience of the fetus. This study aimed to explore how pregnant women experience the abdominal examination and how the examination affects maternal–fetal attachment.

## Methods

### Study design and setting

This study used qualitative data from participant observation and semi-structured interviews. Fieldwork was conducted between September and December 2020 at the Aarhus Antenatal Midwifery Clinic, Aarhus University Hospital in Denmark. Aarhus University Hospital is a public birth facility with approximately 5000 births annually. Antenatal, intrapartum and postpartum care are provided by midwives, who are the primary health care professionals during pregnancy [16, 17]. In Denmark, health care is tax-financed and includes free antenatal care, fetal ultrasound screening, intrapartum and postnatal care [16]. Maternity care is individualised depending on the situation of the pregnant woman and her partner. Women in vulnerable situations are offered extended service, which is provided by midwives and other specialised health care professionals in a specialised team [17]. The extended service is offered to pregnant women who have or have had health, psychological and/or social challenges or an alcohol or drug abuse problem [17]. The women in this study were recruited from Aarhus University Hospital and received extended service from the specialised team for women in vulnerable situations. The socioeconomic situation and vulnerability varied among the included women. Some women had a psychiatric diagnosis or were in a vulnerable situation at the time of the interview. Others had previously experienced life challenges but were not considered particularly vulnerable at the time of pregnancy and enrollment in this study. Moreover, some women had requested to be referred to the specialised team themselves to receive the extended service. The women in this study, thus, was a very heterogenous group. Apart from more visits at the midwife, the extended service offered to the pregnant women also included longer consultations compared to regular maternity service, which may entail more time for the midwife to perform the abdominal examination and to involve the women. Conducting participant observation in the consultation provided by the specialised team is convenient as there is more time to listen to the conversation between the midwives and pregnant women and to observe their interaction during the examinations.

### Participant observation and consent

Participant observation was conducted at the antenatal midwifery consultation. The overall objective was to gain insight into the midwifery consultation as a setting where the abdominal examination is used routinely. Furthermore, the participant observation was used to qualify the interview guide. Admission to observe and recruit participants was granted from the consultant midwife in charge at the extended midwifery team at the Aarhus Antenatal Midwifery Practice. The consultant midwife organised contact and permission to attend the consultations with the individual midwife in charge of the consultation. Further, permission was obtained from the individual women and any partners before the consultations started. The first author briefly described herself (her education and job position as a researcher), and her role during the consultation. The first author was seated in a chair at a distance from the table with the midwife and the pregnant woman. The first author stayed seated during the consultation to maintain a proper distance as the data collection was performed during the COVID-19 pandemic. The abdominal examination was performed approximately 5 meters away, from where first author was seated. From this position, the first author was able to see the face of the pregnant woman and follow the interaction between her and the midwife and the conversation between them. However, from this position, the first author could not fully observe the midwives’ manual palpation. Notes were written during the midwifery consultation and the first author’s interaction with the midwife and the women in terms of eye contact, smiles and brief small talk appeared to occur only a few times. Thus, the participant observation changed into almost complete observation [18]. Using participant observation provided the first author with important insights into a private space normally reserved for only the midwife, the pregnant woman and her close relatives. In total, 26 consultations were observed in a period from mid-August to end-November 2020.

### Interview participants and recruitment

After each of the 26 consultations observed, the women were invited to participate in an interview. A description of the study was provided to the woman at the end of the consultation. If the woman consented to participate, she was later contacted for an interview. All participants included for interview were recruited after participant observation. Characteristics of the 10 participants are presented in Table 1. Because this study focused on the abdominal examination, we prioritised to observe consultations with women who had experienced the examination more than once and, thus, had more experience to share during the interview. Therefore, most of the interviewed women were in mid- or late pregnancy. In total, 10 women were included for interview in this study.

**Table 1:** Basic characteristics of the participants

	n
Maternal Age^1^ (years)	
20–29	4
30–39	6
Parity	
Nulliparous	7
Multiparous	3
Marital status	
Partner/married	9
No partner	1
Occupation	
Student	2
Employed	6
Unemployed	2
Gestational week^2^	
20–29	3
30–40	7
Number of midwifery consultations	
1–2	3
3–5	6
6–8	1

^1^ Mean maternal age (years): 30,8

^2^ Mean gestational week: 32,2

### Interviews

A semi-structured interview guide was used to conduct the interviews, which ensured that the pre-determined themes in the guide were discussed and also enabled the interviewer (the first author) to follow new directions during interviews when interesting themes emerged. The interviews were conducted individually at the women’s private homes or at Aarhus University Hospital upon the woman’s request. The 10 interviews lasted from 37 minutes to 89 minutes, and all interviews were audio recorded. The interviews were initiated with questions regarding expectations and experiences of a midwifery consultation, followed by questions addressing attachment and the abdominal examination. During the interviews, exploratory and clarifying questions were used to explore the women’s experiences in depth. Specific observations from the consultation were referred to during the interviews, for example, “During the abdominal examination, I observed the midwife occasionally invites the woman to participate in palpating the abdomen. Have you tried this?”. This type of question was used to facilitate focus on the experience of the abdominal examination.

### Thematic analysis

The analysis started during the data collection process where the exploration of initial themes where pursued inspired by perspectives from existing literature on pregnancy embodiment ([19–22]). Inspired by Braun and Clarke’s [23] description of reflexive thematic analysis, the interviews were transcribed verbatim, and codes, themes and perspectives were continually discussed among the authors. The transcripts were coded and named in NVivo 12 Pro by first author using an inductive approach, and long text extracts were identified and selected to maintain the context. Themes were developed and named by the authors in an iterative process refining each theme. The presented themes were included because they were pertinent and recurring across the interviews. The field notes from participant observation with a focus on the abdominal examination were short and not coded but studied during the analysis.

### Ethics

Information about the study was provided to the women at the midwifery consultation to consider participation in an interview. All participants were provided with written information and needed to consent before the interview and further assured anonymity. However, they were informed that they might be able to recognise their own quotations in the publication. The participants’ real names are known to the authors. For ethical reasons, the first author did not participate in consultations if she personally knew the pregnant women. During the fieldwork, situational ethical cases arise as a result of the social interactions that are necessarily involved in qualitative research [24]. As an example, two women became emotionally affected during the interviews and the first author offered the women a break before the interview continued. The women were further advised to contact the interviewer if they had any questions following the interview and to contact their midwife if they asked the interviewer for professional issues.

## Results

During the analysis, the following four themes reflecting the women’s experiences of the abdominal examination were identified as central: *an essential examination*, *the baby becomes real*, *the importance of being involved* and *different senses provide different experiences*. The context for these themes was the extended midwifery consultation, exemplified in the following by the case of Ida:

At the antenatal clinic, a smiling woman entered the room. A welcoming midwife greeted her, and the woman sat down at the round table facing the midwife. The midwife started the conversation, “Hi Ida, how are you?”. Ida talked about her well-being, focusing on the limited sleep she has experienced during the night, her aching back and that she was looking forward to her upcoming maternity leave. The midwife listened and provided advice concerning Ida’s well-being. The midwife directed the conversation and looked several times at Ida’s papers placed in front of her, focusing on subjects to be followed-up from their last meeting. In addition to her physical well-being, Ida’s relationship, mental health and expectations for her future life were addressed before the midwife prepared her for the abdominal examination. The midwife instructed Ida to lay on the examination couch, “Now we will examine the little one”. Ida laid on the examination couch and pulled up her shirt to reveal her pregnant abdomen. The midwife used hand sanitizer and said warningly, “I may have cold hands”. Ida smiled at the midwife and replied, “It’s okay.” before the midwife placed her hands on Ida’s abdomen. The midwife asked, “Do you feel movement from the baby?”. Ida replied, “Yes. So much that it’s almost annoying. But it’s also nice”. The atmosphere was positive and joyful. The midwife said, “Let’s see how the little one is positioned”. The midwife examined Ida’s abdomen using her hands while Ida focused on the ceiling. Finally, the midwife revealed, “Here is a small baby head and his back is at this side.” pointing at the right side of Ida’s abdomen. The midwife then estimated the weight of the baby and assured Ida that “He is growing fine.” and asked if she had the same feeling. The midwife measured the symphysis-fundus distance asking if Ida and her boyfriend had decided a name for the baby yet. The midwife applied gel on Ida’s abdomen and searched for the baby’s heartbeat using a doptone. The sound of a fast heartbeat emerged immediately. The baby was moving in the abdomen and the sound of the heart beating fast was evident. The midwife started rocking her head in pace with the sound of the heartbeat and Ida smiled at her. The midwife assured Ida, “Very fine, beautiful! The little one is doing well.” while Ida removed gel from her abdomen and climbed down from the couch.

### An essential examination

The case describes a typical abdominal examination at the antenatal midwifery clinic. During participant observation and interviews, it was clear that the women expected midwives to perform the abdominal examination because it was a fundamental part of a midwifery consultation for them. One of the women, Christina, narrated how she would find it strange if the examination was not a part of the consultation:

*That’s why you are there. It’s because they have to feel you [your abdomen] and she [the midwife] has to find out his position, and well yes, I think it would be strange otherwise. (Christina).*

Another woman, Clara, shared this opinion because she considered the abdominal examination as the main purpose of a midwifery consultation:

*That’s what I kind of find most important, to get that physical check-up, you know. And well, eh, to find out if everything is fine. So, well it’s my main purpose when seeing the midwife, it’s to get the abdominal palpation. (Clara).*

The examination had an implicit acceptance between the midwife and the women, who took it for granted that the examination would be performed at every consultation. This was clear during participant observation wherein the women without any hesitation laid on the examination couch and uncovered their abdomen as the midwife started preparing for the examination. Helene, another participant, stated, “That’s what midwives should do”.

### The baby becomes real

The women mentioned the abdominal examination as the moment when they imagined their baby most intensively during the consultation with the midwife. During the examination, the baby became real in their minds, especially when the midwife described and mentioned the baby as she conducted the palpation of the woman’s abdomen and estimated weight and felt body parts of the baby. The examination was described as “specific”, “tangible” and “hands on”. Anna expressed that the examination made her imagine her baby:

*You get a stronger feeling that there is a baby inside your abdomen because, well “around here is the back and here is the head and it is about this big and the little butt” and so. You think more that it’s not just something moving, something fluffy, it’s actually a baby. So, it makes sense that there are legs kicking you here and so, yes. (Anna).*

As narrated by Anna, it became easier for her to visualise the baby inside her abdomen when the midwife was touching her abdomen and describing the presence of the baby. Ida also described that she felt reassurance that the baby was real and not just something that she was imagining when the midwife touched her abdomen and involved Ida during the examination. For Ida, her baby became tangible during the examination. She explained how it made her start thinking about her baby and reminded her that her baby actually existed. Ida further mentioned that she was able to visualise the silhouette of her baby when the midwife outlined the baby’s body while palpating Ida’s abdomen gently during the manual examination: “I can see it when she presses down. She presses [my abdomen] all the way down and around me, so that means you kind of get the whole silhouette of the baby”. In this way, the midwife became a facilitator for the women to visualise their baby and confirm the existence of the baby. Moreover, it was not only during the abdominal examination that the midwife was able to facilitate the women’s perception of their unborn baby. When the midwife talked and asked about the baby, some women described how they felt reassurance that their baby was real. Thea narrated how she felt when the midwife started talking and asking about her baby:

*In a way it’s nice, but it’s also a little surreal because I still do not quite feel that it is a real human being coming out even though I’ll be giving birth soon. (…*) *I am just running around, and she kicks in the abdomen. I try to talk to the abdomen and try to remind myself, there is a human being in there, but sometimes you forget that oops, it’s not just my abdomen rumbling. Well, so it becomes more obvious, I am reminded when the midwife is asking about her. (Thea).*

Apart from palpating the woman’s abdomen during the examination, the midwife also auscultated the heartbeat of the baby using a doptone, allowing the woman to hear the heartbeat of her baby. This part of the abdominal examination was important for most women. Listening to their baby’s heartbeat had a positive impact on the women. Martha described her feelings:

*It gives me peace in my body, you know. Peace of my mind to feel that it is okay today – I can, well, relax until next time or relax when I walk out of the door and I have such a happy feeling. Okay, this was nice. There is still something in there. (Martha).*

To be able to hear the heartbeat provided her peace and assurance that her baby was okay. Another woman, Heidi, described the heartbeat as “( …) the best sound in the whole world” and she further described her feelings:

*I get an exceptionally great feeling of joy inside and I am filled with love and warmth and with a lot of weird feelings. Happy feelings you know and love and it’s a new situation, but it’s exciting. (…) I think it’s totally amazing and I can’t help but smile all over my face when I hear it. To me, it’s the most amazing sound in the whole world. Eh, it’s simply the sound of his heartbeat, it’s the most amazing sound. I can, I can’t describe it differently because it is so amazing, and I am, you are just filled with love. (Heidi).*

As witnessed during participant observation, the sound of the heartbeat was experienced as pivotal to the women. When the midwife found the heartbeat using the doptone, it seemed to lead to a feeling of release in the women as most of them reacted immediately when the sound appeared. Some women became emotionally touched on hearing the sound of their baby’s heartbeat and others smiled at the midwife while the baby’s heart was beating fast aloud in the room.

### The importance of being involved

As described above, midwives have immense potential to facilitate the women’s attachment to their babies. However, the nature of the abdominal examination entails a risk for disturbing the potential it possesses. The woman’s body integrity during the examination is important to consider acknowledging that the procedure may be intimate or feel transgressive for some women. Heidi described the first time she experienced the abdominal examination:

*It was a bit like crossing some boundaries, I think. Eh, when you suddenly must fling yourself onto a couch and “then you just pull up your clothes and down your pants”. Well, okay then! Eh, it’s not that nice. But, eh, it serves a good purpose. (Heidi).*

Thea experienced the examination as intimate the first time it was conducted because the midwife touched her pubic bone, for which she was not prepared. Some women described the examination as unpleasant when the midwife applied manual pressure to certain areas, such as the pubic bone, that are sore or hurtful owing to pregnancy discomfort. To be able to perform the abdominal examination, the midwife asked the women to relax their abdomen. However, Anna mentioned that she always had issues with others touching her abdomen, which resulted in her body being tense. Therefore, it was difficult for her body to be sufficiently relaxed during the abdominal examination, thereby making the examination uncomfortable. Nevertheless, Anna explained that she could not imagine a consultation at the midwifery clinic without the abdominal examination:

*No. I could not. Well, because you also want to know that he is in the right position. So, in that way I actually think it’s good that it’s [the abdominal examination] performed. I just have to cross my own boundaries. (Anna).*

Anna described the examination as “( …) a very ambivalent experience” because on one hand, she finds it uncomfortable but on the other hand, she appreciates the examination as it provides her with valuable attention and attachment towards her baby.

Being prepared and involved before and during the examination are significant for how the abdominal examination is experienced by the women. In this study, the women expressed that they found the examination to be a positive experience when the midwife prepared and involved them in the examination and clarified what she was doing while palpating their abdomen:

*Last time when she was feeling his head – it, eh, hurt a bit. But she said it from the start. They are very good at telling you that it might hurt a bit. (Christina).*

The importance of being informed during the abdominal examination was highlighted in some women’s descriptions of the examination. Being involved during the examination may, thus, be of major importance for how it is experienced. One woman described that she preferred to be involved and further asked where she felt the baby when the midwife examined the position of the baby:

*I like when she asks if I can feel his position, eh, in there. Eh, also in relation to bonding, I can feel he is lying with his head down or something. Eh, but it’s also, so you can try to sense his position by yourself and then be confirmed. How good is my own feeling of this. (Anna).*

As Anna explained, another aspect of being involved during the examination was to support her own intuition and imagination of her baby, which she described as being important in relation to the bonding process between her and her baby.

During the examination, some of the midwives invited the women to participate during the palpation of the abdomen, which induced an immediate obvious excitement in the women. In one of the examinations observed, the midwife asked the woman if she would participate in palpating the baby. The midwife and woman performed the abdominal palpation together using their twinned hands. The woman obviously became excited and bursting out “wow”. During another consultation, the midwife invited the woman to palpate the head of her baby in the pelvic inlet and the woman’s reaction was “Oh my god! Is that her head?” This obvious excitement experienced among the women was induced by the midwife involving the women physically in the examination, thereby stimulating the woman’s own sense of touch and realisation of their baby. Besides being able to check her own sensation of where she felt her baby, Anna reflected on the importance of being involved in the examination in relation to her own self-esteem:

*That it’s not just something, she just does to me and then “tap, tap, tap”, then it’s over, eh, and he lies like this and like that and he’s about this big (…) Well, I think, I would feel it was, well, a little more transgressive, because then I think it would feel like she forgot a bit that it’s my abdomen. Eh, that it’s not just a child you just have to examine. (Anna).*

During the interviews, some women referred to the approach of another health examination in maternity care, the fetal ultrasound screening. Similar to the abdominal examination, this procedure requires the pregnant woman to bare her abdomen so it can be the examined using the ultrasound transducer. Thea reflected on the way a health professional performing the ultrasound examination almost pulled her pants down for her before examining her abdomen:

*Some of the people who do the scan, sometimes they’re well, they almost pull them [the pants] down for you. Instead of me pulling them down, you know. Because you’re just another patient to them. I feel that I would like to be allowed, you know, to do that myself. (Thea).*

Even though Thea did not experience any of the midwives doing this during the abdominal examination, it underscores the importance of engaging the women before and during the examination, thereby ensuring the respect and autonomy of the women. The risk of women feeling reduced to just a body part, in this case an abdomen, is further described below:

*I think that, well, maybe it’s also because sometimes it goes a little too fast because people are waiting or I don’t know, but anyway, I feel sometimes that they forget that I’m not just a body lying here [laughing]. I think it’s nice to pull down my trousers myself and like, “well, now I’m ready”. (Thea).*

However, Thea did not experience the same practice at the midwifery consultations:

*“It’s more about seeing the human being in a way. Where I feel at the ultrasound examinations, well it’s more about the scanning, I think” (Thea).*

### Different senses provide different experiences

During the interviews, the women’s experience of the ultrasound examination was discussed. This provided insights about the abdominal examination compared to the ultrasound examination, which provides the women with a visual representation of their baby. While the abdominal examination provides the women with the sense of touch via the midwife’s hands and thereby stimulates their own feeling of their baby, the ultrasound examination provides the women with another sensual aspect, the sense of sight, which is also perceived as important by the women. The women in this study described feeling excited for the ultrasound examination, because it was important for them to be able to see their baby. Heidi explained:

*“Eh, it means a lot to me, eh, mentally to be allowed to see him and hear him and see him moving and not just to feel it”. (Heidi).*

The women described that the visual aspect of the ultrasound examination enabled them to imagine their child better. However, the abdominal examination is crucial to the women because it provides them with important aspects that are gained from the touch of the midwife’s hands, which the ultrasound examination does not provide. Some women described a feeling of being distanced from the ultrasound screen, which was not a part of their body. They found it hard to relate to a screen away from them during the ultrasound examination. However, when the midwife touched their abdomen and involved them, the experience became more real and relatable:

*It’s more right here that it’s happening, you know. I think the ultrasound examination can feel like, eh, okay I can see my child on the screen there, but it’s far away in some way... The [abdominal] examination is more, well, then we are feeling my abdomen and I can feel it is my body being touched and it’s kind of, well it gives a little more, this is where it happens and not on a screen or as a description on a piece of paper. (Ida).*

Similar to Ida, Thea also described a feeling of being distanced from the ultrasound screen and the experience as “( …) something going on at the screen rather than something going on in real life”.

The ultrasound examination is regarded important as it visualises the baby. However, because the image is presented on a screen away from the woman’s body, it can be experienced as a distanced snapshot compared with when the midwife is palpating the abdomen and may be even involving the woman in the examination, thereby providing an embodied experience. Hence, the two examinations contribute with two relevant but different insights for the women. The ultrasound examination allows the women to see their unborn baby themselves thereby supporting the women’s visual sense. The abdominal examination supports the sensory aspect of the bodily sensation of the baby inside the woman’s abdomen, thereby stimulating the process of bodily attachment. Anna also described another important aspect of the abdominal examination:

*Well, it’s good that someone touches you and that you don’t have to see it. Eh, mostly for, you know, learning. It has been a big thing for me, this trusting my body, you know. […*] *The screen is kind of. It’s away from you, right? And there’s no, well yes, you can see it, but it’s a little harder to believe it. Eh, instead of someone who just kind of pushed him a little around. […] I often find it a little harder to identify myself with that screen. (Anna).*

As Anna described, the abdominal examination helped her attain a better sensation of her own body. It was easier for her to believe that she had a baby inside her abdomen when the midwife “rummaged” around with her baby. Thus, when the midwife is performing the abdominal examination, she is supporting the woman’s attachment to their baby who becomes relatable and tangible and something that is within the woman’s body as opposed to the ultrasound screen that is distanced from the woman.

## Discussion

In this study, we described how pregnant women experience the abdominal examination in antenatal care. Most women underscored the manual palpation and auscultation, as the central and obvious component of a midwifery consultation, which provided feelings of reassurance and security regarding their baby’s and their own health. This finding aligns with those of a Swiss study of pregnant womens’ attitudes towards surveillance medicine, reporting some women appreciated pregnancy examinations because they helped eliminate uncertainty [25]. In the same line of argument, sociologist Lupton has presented health technologies as methods of “taming uncertainty”, which render the body visible and knowable in biomedical terms [26].

Apart from gaining reassurance from the midwife, the women in this study emphasised great appreciation towards the embodied experience of the abdominal examination as a process of making sense of and relating to their bodily sensations of their baby’s movements and positioning. This finding is consistent with a Canadian study where the transition to motherhood is experienced as a process shaped by sensorial experiences of pregnant embodiment [19]. Furthermore, the embodied experience of pregnancy and the sensorial experience of different types of examinations may be understood in relation to cultural meanings of the senses. In the western culture, the sense of vision has for long been ascribed with great significance to the acquisition of knowledge [27, 28]. In maternity care, this has entailed that visual representations of the fetus using ultrasound examinations have become central to diagnostic practices as well as the social construction of fetal personhood [29, 30]. The women in this study expressed appreciation and excitement towards visual representations of their baby, accompanied by the sound of fetal heartbeat. This finding aligns with those of the historian, Duden [20], who argued that with the use of modern medical technologies, optic representations have replaced haptic sensations in the process of “knowing” pregnant bodies. However, some women interviewed in this study described experiences of ultrasound examinations as distanced or less relatable than the midwife’s abdominal examination wherein touch was experienced via her manual palpation.

In a study of Mexican maternity care, anthropologist Howes-Mischel [31] suggested that sound via audio fetal heartbeat technology, in addition to sight via ultra-sonography, is a potent tool for constructing fetal personhood, and midwives work as facilitators of the social process of a mother being acquainted with her fetus. Our findings add to those of Howes-Mischel, indicating that the process of a pregnant woman gaining acquaintance with her unborn baby can also emerge through the practice of manual palpation. As women in this study narrated, the abdominal examination can encompass both the midwife’s reassuring explanations regarding the baby, for example the baby’s growth and positioning, as well as her affirming inclusion of the mother’s own internal sensations. The finding regarding the women’s experience of being acquainted with their unborn baby through haptic sensations is supported by findings in studies of pregnancy embodiment [19, 32] and underpin the sensorial interaction between a pregnant woman and the fetus as essential to the process of bonding. This study points to the perspective that the abdominal examination has the potential to reinforce this process.

The women in this study underscored the need for involvement and information before and during the abdominal examination. Involvement and information made the experience of the examination more positive, which might be a better foundation for the women to realise the full potential offered by the abdominal examination to enhance maternal–fetal attachment. Although the women in this study expressed their appreciation of the abdominal examination and expected it to be performed at each midwifery consultation, it also induced some discomfort in some women. Thus, some of them argued that the performance of the abdominal examination, if not conducted respectfully and with compassion, might disrupt the experience and potentially be perceived as transgressive and ambivalent. These findings align with the arguments from a body of literature on pregnancy risk discourse, pointing to how modern medical technologies and examinations targeted at pregnant women may turn over the control and interpretation of the pregnancy from the pregnant women to the medical specialists [21, 22, 33, 34]. In this study, some women narrated their experience of being reduced to “an abdomen” when they were not respectfully addressed and involved during other pregnancy examinations. However, in their accounts of abdominal examinations, feelings of risk and disembodiment were not characteristic in their experiences. Instead, with the midwife providing a guided touch and physically involving and vocally informing them, most women narrated gaining an occasion to appreciate the direct, bodily connection with their baby. A meta-ethnographic study on the experience of touch across health care professions suggested that touch in health care contexts holds the potential for communicating care “above words” [35]. In alignment with this perspective, our study found that a safe space for experiencing the midwife’s guided touch can be created through subtle initiations and negotiations of physical contact.

Several studies of women’s experiences of diagnostic technology describe the significance of the operator’s or midwife’s feedback [31, 36, 37]. This study underscores the importance of the midwife’s articulated involvement and exchange of information are consistent with those in previous studies. For example, Patel and Rajasingam [38] argued for the importance of user engagement as a “mutual exchange of information between the patient and health professional”. Findings from studies regarding women’s experiences of pelvic examination highlight the importance of continuous information during the examination to regain control [39, 40]. Moreover, the relationship with the midwife is important for the women to feel comfortable before and during the examination. A Norwegian intervention study on continuity in care suggested that a trusting and supporting relationship between the mother and midwife had a positive impact on the mother’s experience of pregnancy and childbirth [41, 42]. Furthermore, Hopkins et al. [43] reported that interventions to increase social support and decrease anxiety can enhance maternal–fetal attachment.

In a sociological study examining how relationships are established through health care technologies, Pols and Moser [44] argued that the relationships between people and technologies within different practices can enable different affective and social relationships. In the light of this perspective, this study indicates that a pregnant woman’s experience of manual palpation and auscultation is both dependent on her interaction with the midwife and influential to the relationship with her unborn child. The interviewed women narrated how the midwife, when performing abdominal examination, could enhance the experience by guiding the mother’s touch and showing interest in the mother’s own haptic impressions. Thus, our findings suggest that the midwife, during the abdominal examination, can be a facilitator for the pregnant woman in the prenatal process of realising, haptically sensing and, thus, gaining acquaintance with her unborn baby.

Even though all women included in this study were those who received extended service from the specialised team for women in former or current vulnerable life situations, we argue that the findings of this study can be generalized to all pregnant women. All women interviewed in this study reported similar experiences regardless of their socioeconomic background or vulnerability status, which indicates that the insights from this study may be of relevance not only for the women enrolled in the extended service but also for women in antenatal care in general. However, future research among diverse populations of pregnant women could prove to be interesting and add further important insights to the findings from this study.

## Conclusion

Our study contributes with novel insights into how the abdominal examination performed by midwives is experienced by pregnant women. The abdominal examination is regarded as essential by the women as it provides them assurance regarding their baby’s and their own health and well-being. The examination does not only serve as a medical or diagnostic examination as it holds a major potential for reinforcing the pregnant women’s bodily sensations of her baby through the midwife’s manual palpation and auscultation. This indicates that the midwife’s position as the primary health care professional during pregnancy and her professional ability to perform the abdominal examination provides her a profound potential to facilitate maternal–fetal attachment. It should be recognised that touch and haptic sensations, in line with the sense of vision, can be a way for a pregnant woman to become acquainted with her unborn child. Another important finding in this study is the importance of information and involvement before and during the abdominal examination. By physically and vocally involving the women, the midwife may be able to reduce the discomfort that some women experience during the examination, providing more attention to the potential to facilitate maternal-fetal attachment. Thus, the potential of the abdominal examination to facilitate maternal-fetal attachment is dependent on the interaction between the midwife and mother.

## Supplementary Information

**Additional file 1.** Interview guide.

## Data Availability

The datasets generated and analysed during the current study are not publicly available due to ethical considerations of personally identifiable information, but data are partly available from the corresponding author on reasonable request.
