# Supplementary material for: Abdominal examination during pregnancy may enhance relationships between midwife, mother and child: a qualitative study of pregnant women’s experiences
Source: BMC Pregnancy Childbirth. 2023 Jan 31;23:84. doi: 10.1186/s12884-023-05392-0 (PMC9887567; doi:10.1186/s12884-023-05392-0)
Supplement: Supplementary file 1 — Additional file 1. Interview guide. [file 12884_2023_5392_MOESM1_ESM.docx]

Interview guide

| **Research topic** | **Questions** |
| --- | --- |
| Context | - Please tell me about yourself? |
| Expectations | - What expectations did you have before you started going to the midwife? |
| Experience | - Can you describe the first time you were at a midwifery consultation? - Can you describe how you experience going to a midwifery consultation? - Are you nervous, excited? If so - why? - When is it best to be at the midwifery consultation? - When is it hardest/toughest/most difficult during a midwifery consultation? - How do you feel when the midwife asks you about personal issues? - What is most important during the midwifery consultation? |
| Attachment/  bonding | - What is your experience when the midwife asks about your baby? - When do you think most about your baby during the midwifery consultation? - Does the midwife help you to better imagine your baby/ get a stronger connection to your baby? - When? How? - Is there anything that makes it difficult to think about your baby during a midwife consultation? - When? How? - Do you use your experiences from the midwifery consultation to support your relationship with your baby? - When do you feel the greatest connection to your baby? |
| Palpation | - One of the examinations performed by the midwife is the abdominal examination. Can you describe how you experience the abdominal examination? - How do you experience being palpated on your abdomen? - How was your experience of the first abdominal examination? - Did you feel it was transgressive? Why / why not? Were you nervous? - Could you imagine a midwifery consultation without having an abdominal examination? - Could you imagine that the abdominal examination was replaced by an ultrasound scan? Why / why not? |
| Prenatal bonding | - What does the abdominal examination mean to you? - What does the abdominal examination mean to you in terms of being able to imagine your baby? - Does it make you think more / less about your baby? - What thoughts do you have about your baby during the abdominal examination? - During the abdominal examination, the midwife asks you about your baby: does he / she kick, how he / she is lying, etc. Is it easier to imagine your baby when the midwife asks these questions while she palpates? - How do you experience listening to your baby’s heartbeat during the abdominal examination? - Do you experience any barriers in relation to talking about your baby during the abdominal examination? - What could the midwife do to support your connection with your baby during the abdominal examination? |
